# Angiogenic effects of cell therapy within a biomaterial scaffold in a rat hind limb ischemia model

**DOI:** 10.1038/s41598-021-99579-0

**Published:** 2021-10-15

**Authors:** Saeede Amani, Rasoul Shahrooz, Rahim Hobbenaghi, Rahim Mohammadi, Ali Baradar Khoshfetrat, Ali Karimi, Zahra Bakhtiari, Ian M. Adcock, Esmaeil Mortaz

**Affiliations:** 1 Department of Histology and Embryology, Faculty of Veterinary Medicine, Urmia University, Urmia, Iran; 2 Clinical Tuberculosis and Epidemiology Research Center, National Research Institute of Tuberculosis and Lung Diseases, Shahid Beheshti University of Medical Sciences, Tehran, Iran; 3 Department of Veterinary Pathology, Faculty of Veterinary Medicine, Urmia University, Urmia, Iran; 4 Department of Veterinary Surgery, Faculty of Veterinary Medicine, Urmia University, Urmia, Iran; 5 Chemical Engineering Faculty, Sahand University of Technology, Tabriz, Iran; 6 National Heart & Lung Institute, Imperial College London, London, UK; 7 Department of Immunology, Faculty of Medicine, Shahid Beheshti University of Medical Sciences, Tehran, Iran

**Keywords:** Biotechnology, Cell biology, Immunology, Molecular biology

## Abstract

Critical limb ischemia (CLI) is a life- and limb-threatening condition affecting 1–10% of humans worldwide with peripheral arterial disease. Cellular therapies, such as bone marrow-derived mesenchymal stem cells (MSCs) have been used for the treatment of CLI. However, little information is available regarding the angiogenic potency of MSCs and mast cells (MC) in angiogenesis. The aim of this study was to evaluate the ability of MCs and MSCs to induce angiogenesis in a rat model of ischemic hind limb injury on a background of a tissue engineered hydrogel scaffold. Thirty rats were randomly divided into six control and experimental groups as follows: (a) Control healthy (b) Ischemic positive control with right femoral artery transection, (c) ischemia with hydrogel scaffold, (d) ischemia with hydrogel plus MSC, (e) ischemia with hydrogel plus MC and (f) ischemia with hydrogel plus MSC and MCs. 10^6^ of each cell type, isolated from bone marrow stroma, was injected into the transected artery used to induce hind limb ischemia. The other hind limb served as a non-ischemic control. After 14 days, capillary density, vascular diameter, histomorphometry and immunohistochemistry at the transected location and in gastrocnemius muscles were evaluated. Capillary density and number of blood vessels in the region of the femoral artery transection in animals receiving MSCs and MCs was increased compared to control groups (P < 0.05). Generally the effect of MCs and MSCs was similar although the combined MC/MSC therapy resulted in a reduced, rather than enhanced, effect. In the gastrocnemius muscle, immunohistochemical and histomorphometric observation showed a great ratio of capillaries to muscle fibers in all the cell-receiving groups (P < 0.05). The data indicates that the combination of hydrogel and cell therapy generates a greater angiogenic potential at the ischemic site than cell therapy or hydrogels alone.

## Introduction

Critical limb ischemia (CLI) is the most advanced stage of peripheral arterial disease (PAD) in man^1^. CLI patients have inadequate perfusion leading to pain at rest and tissue loss with amputation rates of 30% at 1 year^2^. There is an increasing incidence of PAD worldwide^3^ and it is estimated that the incidence of CLI will increase substantially in the future^4^ as most patients with CLI do not have appropriate anatomy or conduits for revascularization^5,6^. CLI has a high mortality rate^1,7^ and novel treatments such as cellular therapy are required^8^. Cellular therapies can improve limb salvage by preventing major amputation in CLI patients^9^.

Mesenchymal stem cells (MSCs) are candidates for CLI and promote angiogenesis and arteriogenesis through stromal and paracrine activities^10,11^. MSCs reside in the bone marrow stroma and could be considered a source for autologous cell-based therapy due to their highly proliferative and self-regenerative capability and low immunogenicity^12,13^. MSCs can form capillary-like structures in vitro^14^ but have inadequate retention and viability after delivery in vivo which limits their use in myocardial ischemia, heart failure, cerebral disease, or renal failure^1^.

Mast cells (MCs) increase the proliferation and transmission of MSCs and prevent their differentiation into fibroblasts^15,16^. MCs^17,18^ and MSC^14,19^ are located at or near the site of capillary germination indicating a possible relationship between angiogenesis and these cells^19,20^. MSCs can differentiate into capillary blood vessels in ischemic areas^21^ whilst MCs secrete cytokines and chemokines that enable the migration and differentiation of cells within the ischemic site such as vascular endothelial growth factor (VEGF), basic fibroblast growth factor (bFGF), transforming growth factor beta (TGF-β), tumor necrosis factor alpha (TNFα) and interleukin-8 (IL-8)^18^. For example, bFGFs interfere with the migration, proliferation and differentiation of endothelial cells^22–24^ and enable vascular smooth muscle cells to develop into vessels^25^. In addition, the VEGF family enhance vessel permeability and diameter^24,26^.

In cell therapy, more than 90% of the injected cell suspension is lost and does not engraft^27^. Most studies support the concept that tissue engineering using a three dimensional (3D) porous scaffold improves cell engraftment by controlling cell attachment, mechanical support and the stimulation of new in vivo tissue growth^27–30^. However, the application of synthetic and chemical materials increases the risk of damage to cells and living tissues. Therefore, the importance of natural scaffolds has been highlighted^27^. A key selection criteria for scaffolds is a 3D scaffold with a proper porosity which does not interfere with angiogenesis^27^. Natural polymers such as alginate and gelatin have no impact on angiogenesis^31^ and are cost-effective^29^. The combination of alginate and gelatin has a similar extracellular matrix (ECM) composition to that seen in animals^31^ making the combination an appropriate material for tissue engineering.

We hypothesized that applying MSCs or MCs alone or in combination will enhance the development of angiogenesis under ischemic condition. Thus, we evaluated their therapeutic effects as an add-on cell therapy in a rat model of hind limb ischemia (severing of the femoral artery) on a background of tissue modeling and bioengineering using a hydrogel scaffold.

## Materials and methods

### Study design and animals

Thirty healthy mature male Wistar rats with weight of 200–250 g, were obtained from the animal house, Faculty of Veterinary Medicine, Urmia University, Iran, and randomly divided into six experimental groups (n = 6 per group). Rats were kept in the lab 6 week before the experiment began at an ambient temperature of 23 ± 3 °C, stable air humidity and a natural day/night cycle (14 h light and 10 h darkness). Groups were: (a) Control healthy group; (b) Ischemic control group with ischemia created in the hind limb by femoral artery transection between two ligatures 5 mm apart; (c) Hydrogel scaffold (50 µl) control group; (d) MC injected group; (e) MSC injected group and (f) MCs and MSCs (Mix) group. Hydrogel (alginate-gelatin) (50 µl) scaffold alone or impregnated with 10^6^ of each cell type was applied to the site of ischemia (artery transection). Cells were added to the hydrogels on day 14 after surgery. All procedures were performed based on the guidelines of the Ethics Committee of Urmia University-Iran (Reference No: AECVU-185-2018). The experimental protocols were approved by the Urmia University-Iran ethical committee. The in vivo animal experiments study was carried out in compliance with the ARRIVE guidelines (https://arriveguidelines.org).

### Surgical procedure

The procedure was carried out based on the guidelines of the Ethics Committee of the International Association for the Study of Pain^32^. Rats were anesthetized by intra-peritoneal administration of ketamine-xylazine (ketamine 5%, 90 mg/kg and xylazine 2%, 5 mg/kg) and then a 5 mm portion of the right femoral artery was ligated and resected to create the hind limb ischemia model. The proximal branches, superficial caudal epigastric, and the muscular branches arteries and veins were also resected^33^.

### Histological analysis

Animals were euthanized on day 14 using an overdose of ketamine-xylazine (3 × anesthesia dose) and tissue specimens were taken and fixed in 10% formaldehyde^33^. Paraffin sections (5–7 μm) were prepared using a rotary microtome (Microm, GmbH, Germany). Sections were stained with hematoxylin–eosin (H&E) for histomorphometric studies, Masson’s trichrome for collagen distribution, periodic acid–Schiff (PAS) to assess muscle glycogen and with CD34 immunohistochemistry for the analysis of capillary density and vessel diameter at both the transected location and in gastrocnemius muscles. Tissue samples were photographed with a digital camera (Dino-Eye-AM-7023, Taiwan) and analyzed using Dino Capture 2.0 software (Dino-Lite Europe, Naarden, and the Netherlands). In brief, slides were de-paraffinized with xylene and sections rehydrated using an ethanol gradient. The sections were stained with H&E as described before^34^. The number of capillaries and fibers were counted at 5 random 0.0625 mm^2^ areas at 400× magnification and their ratios calculated. For histomorphometric evaluation of fibers, cross-sectional muscles were evaluated as described previously^34^.

### Rat bone marrow-derived MCs cell isolation

Bone marrow cells were immediately isolated from femur and tibia bones from 6 different animals and MCs derived as described earlier^34,35^.

### Flow cytometry

MC were characterized by flow cytometry as previously described^35^**.** Briefly, MCs were harvested after 3 weeks of culture, and washed with cold PBS and the cell-surface Fc receptor was blocked with the 2.4G2 antibody (Beckon Dickenson, San Diego, CA, (USA). Cells were incubated with FITC-conjugated anti-rat FcεRI antibody (BD Pharmingen, Catalog No. 551469, USA) and PE-conjugated anti-Rat *c-kit* (Santa-Cruz Biotechnology; 2B8, catalog No. sc-19619, USA) antibody in 100 μl of PBS for 1 h at 4 °C. Cells were washed with PBS and 10,000 events were analyzed by flow cytometry (FACS Calibour BD, USA) and compared with matched isotype control antibodies.

### Rat bone marrow-derived mesenchymal stem cells isolation and characterization

Animals were euthanized by intra-peritoneal ketamine-xylazine and their hind legs cleanly shaved and prepared aseptically. A skin incision was made and the lateral surface of the femur and tibia exposed. The metaphyseal regions of the femur was cut with scissors and the bones flushed using an insulin syringe with 1 ml of endotoxin-free complete media. The collected marrow tissue was placed in DMEM (Dulbecco’s Modified Eagles Medium media with antibiotics) and the cells centrifuged for 10 min at 320×*g* at 4 °C. The cells were cultured at the ratio of 1 × 10^6^/ml in a 25 cm^2^ flask in complete media (DMEM, containing 20% FBS and 1% antibiotic mixture of 100 IU/ml penicillin, 100 µg/ml streptomycin) as described previously^36^. Non-adherent cells were removed after 72 h of seeding. The media was changed every 3 or 4 days until the cells reached confluence. Trypsin–EDTA was added for 5 min and the suspension centrifuged at 200*g*, 4 °C for 5 min to collect the cells. The pellet was re-suspended in buffer (1× D-PBS containing 2% FBS and 0.05% sodium azide) to a final concentration of 5 ~ 10 million cells/ml on ice. Then the cells were aliquot in 100 μl of FACS buffer before addition of antibodies. Cells were stained with CD54-FITC (cat No. 554969), CD90.1-FITC (cat No. 561973), CD45-PE (cat No. 559135) and CD31-PE (cat No. 555027) and then compared with matched isotype control antibodies (Mouse IgG1 (cat No. 551954) for 30 min at 4 °C. All antibodies were purchased from BD Biosciences, USA. Six MSC lines were generated and used in these experiments.

For analysis, the cells were washed with FACS buffer twice and then suspend in 100 μl cell FACS buffer. 10,000 events were analyzed by flow cytometry (FACS Calibour, BD, USA) and the data analyzed by FLOJo version 8. Results were calculated as percentages within the indicated gates.

### Scaffold

Alginate-gelatin (Sigma-Aldrich, USA, cat No. 9005-38-3) was the main component of the hydrogel scaffold. After alginate modification with gelatin (Sigma-Aldrich, USA, cat. No: 9000-70-8), phenolic groups were added for stability and to control the gelation time as previously described^27–29,31^. The hydrogel (2:1 ratio alginate:gelatin) was then formed by incubation with peroxidase^37–39^.

The hydrogel solution was prepared as previously described^35^. Briefly, 75 μl hydrogel was added to 10^6^ cells before addition of 7.5 μl horseradish peroxidase (HRP) with gentle mixing. The resultant mixture was then implanted at the surgical site and 15 μl hydrogen peroxide was immediately added to the scaffolds in vivo^40,41^.

### Immunohistochemical analysis

Tissue section slides were heated at 60 °C for 25 min in oven (Venticell, MMM, Einrichtungen, Germany). Sections were gently washed in washing buffer and then incubated with an anti-CD34 (a marker of endothelial progenitor cells) primary antibody (1:5000, Abcam, Cambridge, MA, USA, cat No. ab81289) for 15 min. Diaminobenzidine-substrate-chromogen was then added for 5 min before slides were washed and counterstained using hematoxylin for 5 s. Sections were then dipped in ammonia solution (0.037 M/L) 10 times, rinsed with distilled water before being cover-slipped. Positive brown staining was observed under a light microscope. The capillary count was conducted by optical microscope and Dinolite lense digital camera (Dino-Eye-AM-7023) and related software at magnification of 400× at the area of 0.0625 mm^2^. Larger vasculatures were counted at magnification of 100× at the area of 0.88 mm^2^ at each tissue section. 5 samples from separate animals in each group were examined^35^.

### Determination of collagen fiber density

Masson trichrome-stained collagen fibers were visualized by light microscopy (Zeiss, Cyber-Shot, Japan). To assess the collagen intensity in the histological sections, the pixel-based intensity of blue-staining, representing the collagen fibers, was assessed at 2530 µm × 2530 µm sections of a photomicrograph by software analysis (Image pro-insight, Media Cybernetics, USA). For this purpose, 20-megapixels images were prepared by the on-board camera (Zeiss, Cyber-Shot, Japan), then, the area (2530 µm × 2530 µm) was measured by MEZZURE software on the photomicrograph, and the means of pixel-based intensities, obtained from 3 images from a section (in total 15 section/each group), were evaluated. Finally, the mean ± SD of intensities was compared between groups^34^.

### Statistics

The data were analyzed by GraphpadPrism version 8. All values are expressed as mean ± SEM. Differences between experimental groups were analyzed using one-way ANOVA. A Bonferroni test was used to identify significant differences between the different pairs of groups. The level of significance was set at *P* < 0.05.

## Results

### Differentiation of bone marrow cells into Mast cells and mesenchymal stem cells

**Figure 1:**
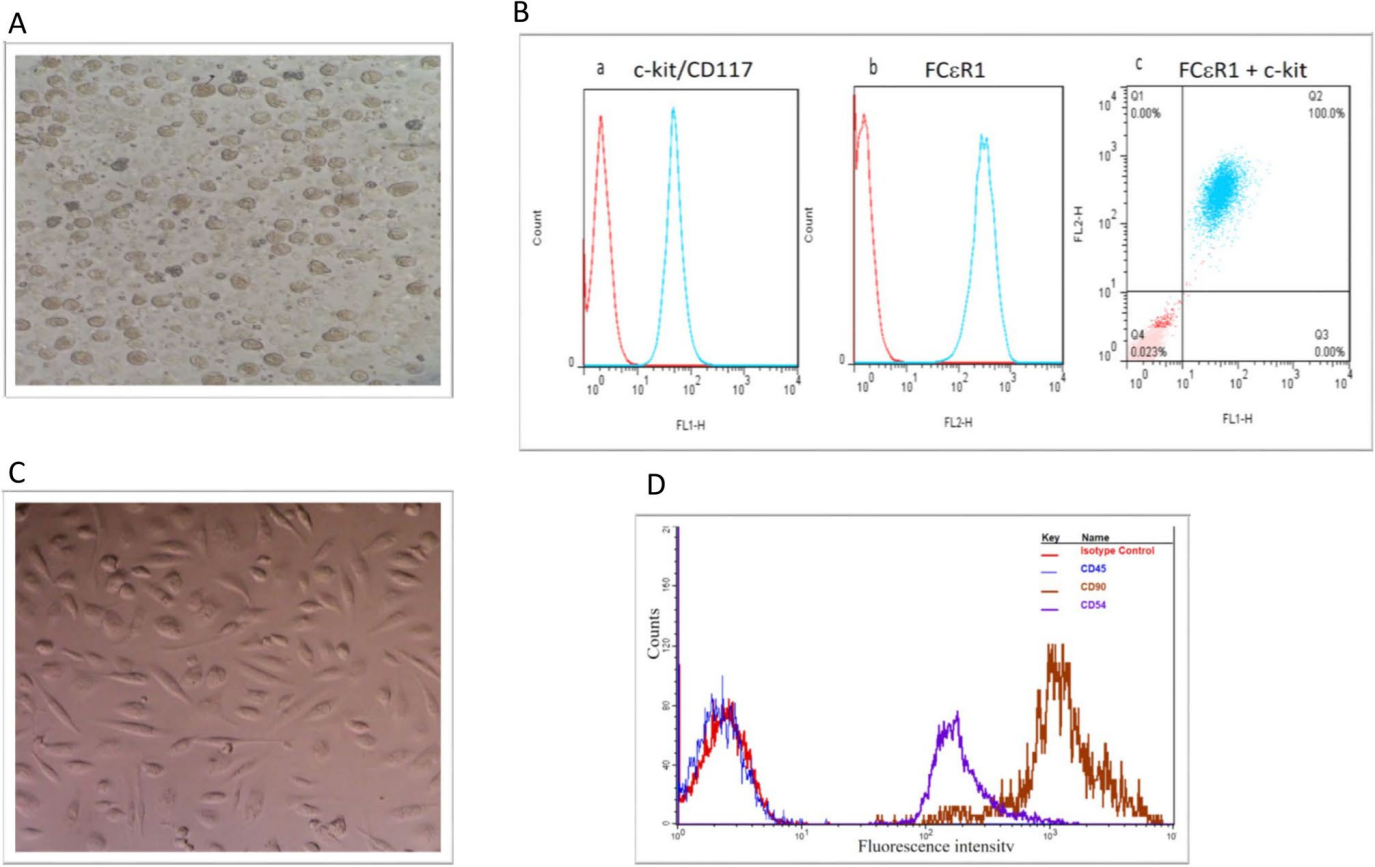
Characterisation of bone marrow-derived mast cells (MCs) and mesenchymal stem cells (MSCs) by morphology and flow cytometry. (**A**) Representative photomicrograph of rat bone marrow cells following culture in complete medium (as described at M&M section) in presence of splenic supernatant of rat pokeweed medium [PWM-SCM, 20% (v/v)] on day 23 (passage 4–5) showing rounded mast cell morphology with intracellular granules. > 92% of cells gave evidence of MC differentiation. Representative flow cytometric analysis of these cells (**B**) demonstrated staining for CD117 (c-kit) (Ba), FCϵRI (Bb) and dual staining for CD117 and FCϵR (Bc). (**C**) Cultured bone marrow cells as described at M&M section after passage 3–4 had the appearance of a single population of adherent MSCs with a spindle-shaped morphology. (**D**) Flow cytometric analysis of confluent MSCs determined the cell surface expression of CD54-FITC and CD90-FITC and no expression of CD45-PE. IgG1 was used as an isotype control. Demonstrated data is flow cytometric analysis of mesenchymal stem cells in one of independent experiment.

MCs were isolated and cultured over time. Upon plating of bone marrow cells, both adhesive and non-adhesive cells were present. The number of cells present reduced after 5–10 days of culture/passage 3–4 and they took on a heterogeneous morphology. By day 23 the cells took on a mature appearance with granules and a round shape (Fig. 1A). Flow cytometry analysis of harvested cells at day 23 identified > 92% of cells positive for c-kit/CD117 (Fig. 1Ba), FCεRI (Fig. 1Bb) and dual c-kit/CD117-FCεRI (Fig. 1Bc) positive cells. For the development of MSCs, bone marrow cells were cultured in media for up to 72 h after cell seeding and removal of non-adherent cells. Adherent cells at confluence (at third or fourth passage) had the appearance of a single population of MSCs with a spindle-shaped morphology (Fig. 1C). Flow cytometric analysis of MSCs show positive staining against the MSC surface markers CD54 and CD90 but negative for CD45 and the isotype IgG1 control (Fig. 1D).

### Preparation and implantation of the hydrogel scaffold

**Figure 2:**
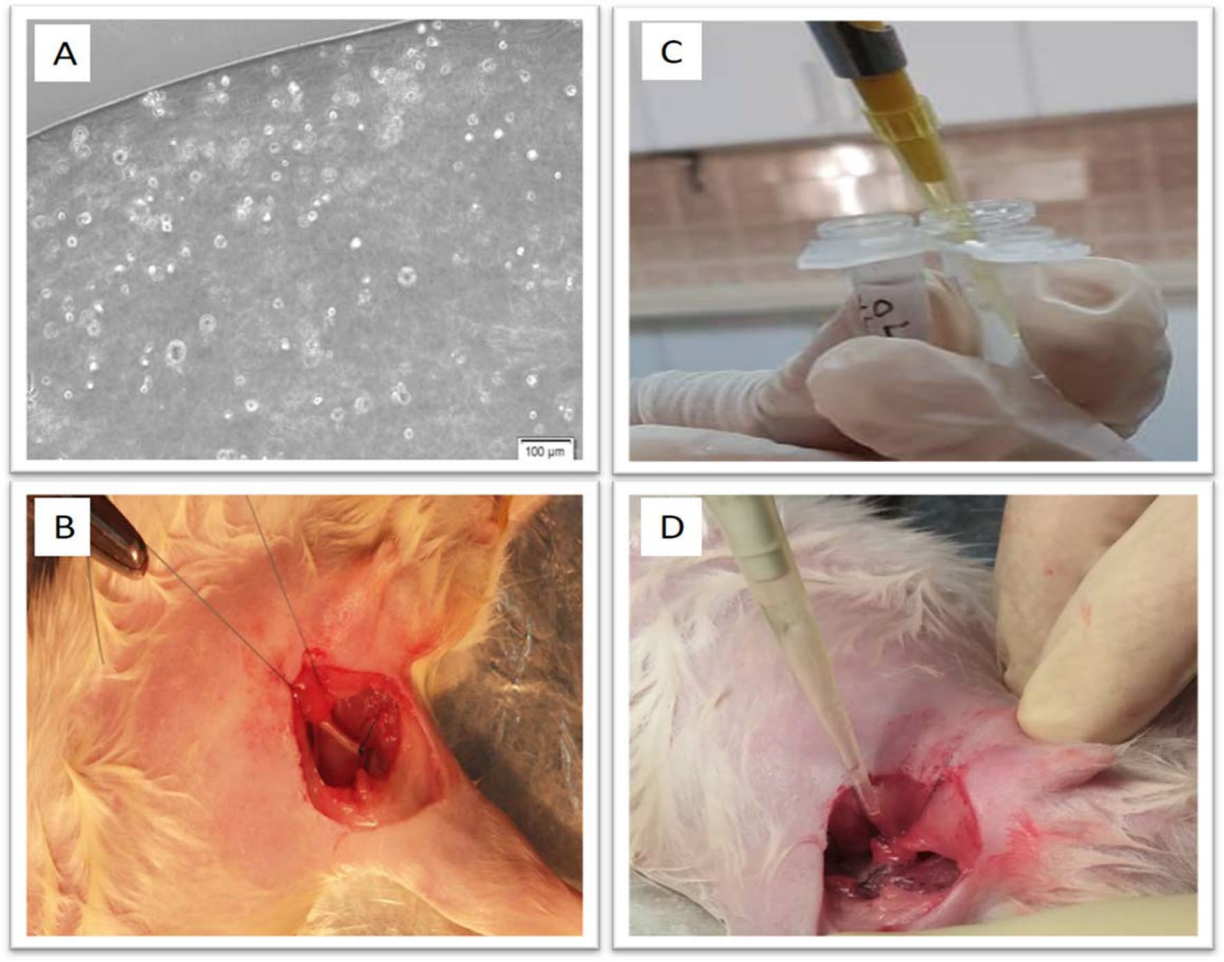
Preparation and implantation of the hydrogel scaffold. (**A**) Representative image showing the morphology and viability of the cells encapsulated within the hydrogel. (**B**) A representative image of the resected area of the femoral artery. (**C**) Photograph showing the addition of 15 μl hydrogen peroxide to the cells encapsulated within the hydrogel**. **(**D**) Representative photograph showing implantation of the resultant mixture into the resected area of the femoral artery.

The hydrogel scaffold was prepared through the main component of alginate-gelatin and then the cells encapsulated by hydrogel solution at density of 10^6^ cells per 75 μl hydrogel (Fig. 2A). The right femoral artery was ligated and resected to create the hind limb ischemia model (Fig. 2B). Hydrogen peroxide was added to the cells encapsulated by hydrogel solution prior to implantation (Fig. 2C). The resultant mixture was immediately implanted into the resected area of femoral artery (Fig. 2D).

### Determination of ischemia

**Figure 3:**
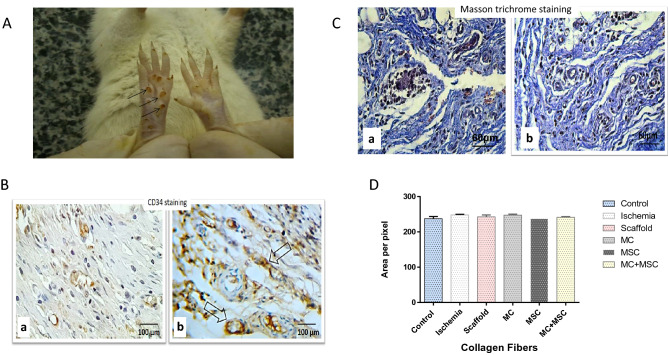
Illustration showing the necrosis in the ischemic left foot pad and histomorphometric analysis of collagen fibers in the femoral artery resected area. (**A**) Representative visual picture of ischemia-induced necrosis (arrowed) of the foot pad. (**B**) Representative immunohistochemical staining for CD34. Endothelial cells appeared brownish-yellow to dark brown with the chromogen. The CD34 endothelial marker demonstrates the intensity of blood vessels in a control animal (a) in comparision to an animal with ischemic tissues where cells were implanted (b). Images are representative of at least n = 5 independent experiment. (**C**) A representative micrograph showing individual morphometric analysis of Masson’s trichrome stained large blood vessels in a control animal (a) compared with an animal with ischemic tissues where cells were implanted. Collagen deposition is detected by a blue color. Images are representative of at least n = 5 independent experiment. (**D**) Histogram showing the mean pixel-based intensities of the blue-staining representing collagen fibers at 2530 µm × 2530 µm a reads of tissue in the different groups. All data are presented in mean ± SEM from 5 animals per group with the left hind limbs acting as a control.

On day 21 after the operation, evidence of necrosis was macroscopically visible in the foot pads and fingers (arrowed area in Fig. 3A). A combination of H&E, Masson’s trichrome staining and IHC for CD34 (arrowed) shows the presence of endothelial cells within capillaries (Fig. 3B). A representative histomorphometric analysis of collagen fiber distribution on tissue vessels is shown (Fig. 3C). There was no significant effect of ischemia or of any treatment on collagen fiber distribution (Fig. 3D).

### Histomorphometry of femoral artery area

**Figure 4:**
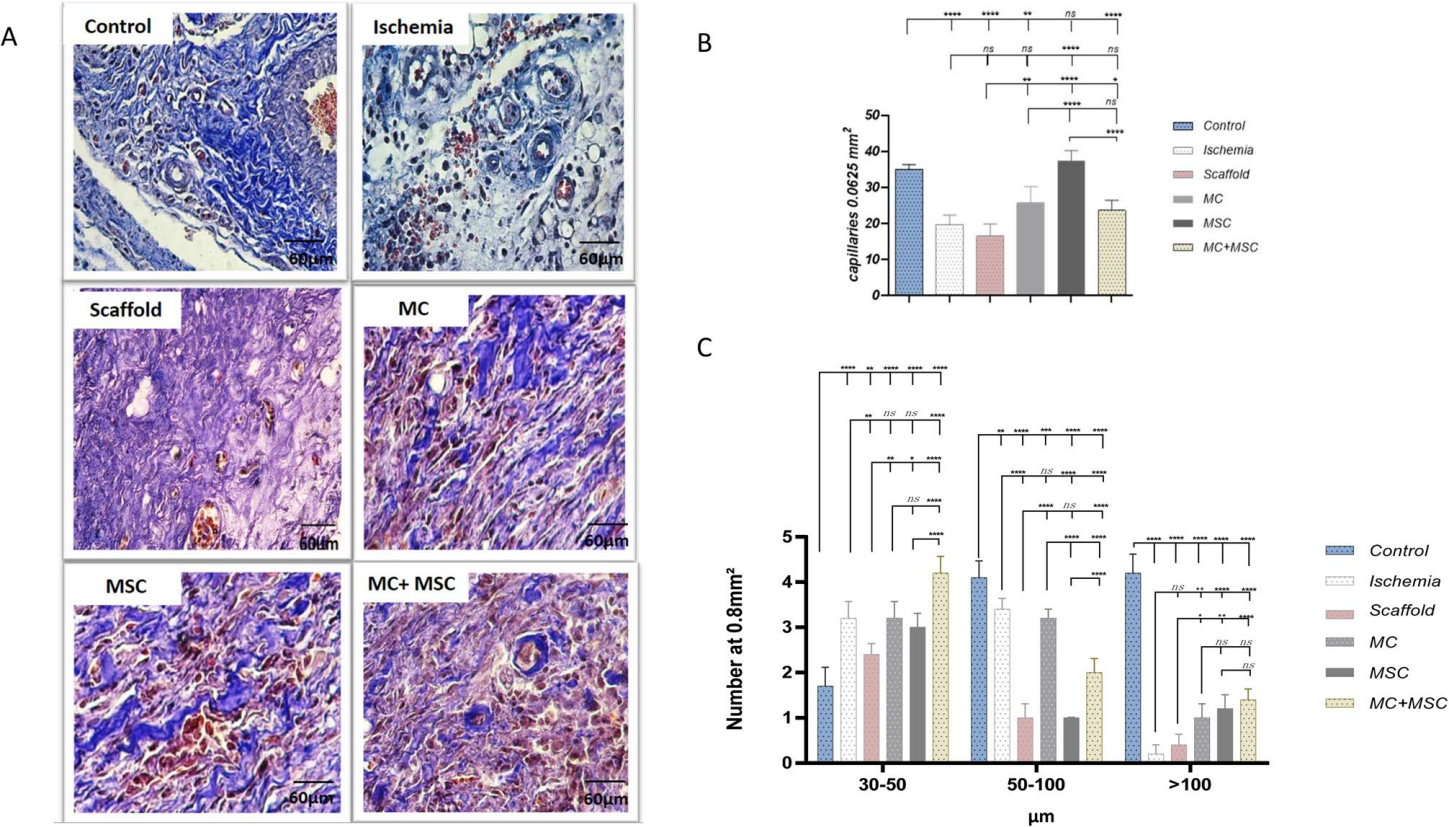
Histomorphometric analysis of capillaries and vessels and qualitative evaluation of angiogenesis in the femoral artery resected area. (**A**) Representative masson trichrome stained sections of the resected area shows increasing angiogenesis in all stem cell treated groups and lack of effect of the hydrogel on angiogenesis. (**B**) Bar graph showing the effect of ischemia on capillary density per 0.0625 mm^2^. (**C**) Histogram showed semi quantitative comparison of blood vessels (intensity and distribution) in femoral artery transected area according to the cross-sectional diameter (30–50, 50–100 and > 100 μm). Data are presented as mean ± SEM of 5 independent experiments. Asterisks indicate statistically significant differences compared to other samples *P ≤ 0.05, **P ≤ 0.01, ***P ≤ 0.001 and ****P ≤ 0.0001.

Qualitative evaluation of angiogenesis in the resected area of the femoral indicated that application of the hydrogel scaffold alone did not stimulate angiogenesis although this was seen with cell therapy (Fig. 4A). In addition, capillaries were counted at the site of femoral artery resection using an optical microscope (magnification 400×) and a graded lens (1.16 mm square mesh size). Ischemia resulted in a significant decrease in capillary density which was significantly reversed in all three treatment groups with hydrogel (*P* < 0.05) (Fig. 4B). Hydrogel/MCs and hydrogel/MSCs groups show a significantly enhanced capillary density compared ischemia and scaffold controls (Fig. 4B). Interestingly, the combined MSCs plus MCs with hydrogel reduced the ability of MSCs to enhance capillary density (Fig. 4B). Based on morphology, we classified the results into 3 groups according to cross-sectional diameter (30–50, 50–100 and > 100 μm) at the site of femoral artery transection (P < 0.05) (Fig. 4C). Ischemia induces a significant increase in small vessels and a reduction in the numbers of medium and large vessels (P < 0.05) (Fig. 4C). The effect of MC and MSC therapy was similar with respect to the formation of small and large vessels (Fig. 4C).

In contrast, MSCs did not affect medium sized vessel formation compared to the hydrogen scaffold. In addition, the combination of MCs plus MSCs enhanced small vessel formation compared to MCs and MSCs alone whilst the combination did not significantly enhance large vessel formation (Fig. 4C). The hydrogel scaffold alone reduced the ischemia-induced enhancement of small vessels and further suppressed the numbers of medium vessels (Fig. 4C). MCs and MSCs significantly enhanced the numbers of small and large vessels compared to the hydrogel scaffold alone whereas only MCs enhanced medium-sized vessel formation with no effect of MSCs (Fig. 4C). The hydrogel scaffold alone had no significant increase in large vessel formation (Fig. 4C).

### Histologic changes in study groups

**Figure 5:**
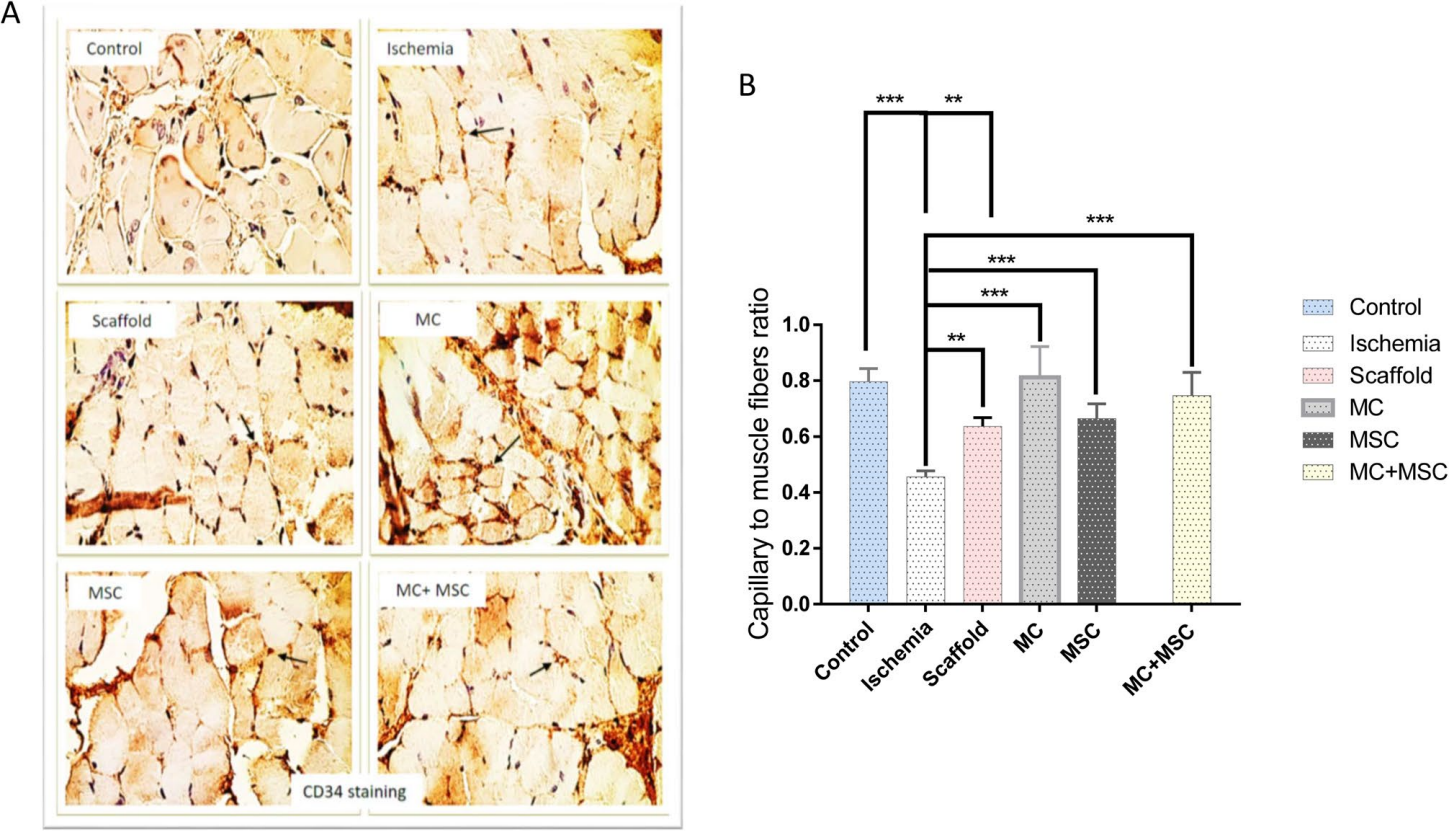
Effect of femoral artery resection and treatment on capillaries to muscle fiber ratio. (**A**) Immunohistochemical staining for CD34 to detect endothelial cells. Positive staining is indicated by the dark brown arrowed area between the gastrocnemius muscle fibers. (**B**) Histogram showing the effect of ischemia and interventions on capillary to gastrocnemius muscle fiber ratio. All values are expressed as the mean ± SEM of results from 5 animals. Asterisks indicate statistically significant differences compared to other samples. *Indicate P value is P < 0.05; **Indicate P value is P < 0.01; *** Indicate P value is P < 0.001.

The numbers of capillaries were assessed by H&E, Masson’s trichrome and CD34 staining. A representative CD34+ endothelial cell within a vessel between the gastrocnemius muscle fibers is indicated by an arrow (1500 magnification) (Fig. 5A). The capillary to muscle fiber ratio was significantly reduced in the ischemia group compared to controls (P < 0.05). This was significantly improved by the hydrogel scaffold but this remained significantly reduced compared to the control levels (Fig. 5B). Although, addition of MCs, MSCs and combined MCs plus MSCs did not significantly affect the ratio compared to hydrogel scaffold alone, these were no longer different from the ratio seen in control animals (Fig. 5B).

## Discussion

In a femoral artery transection model of CLI the application of hydrogel, MCs and MSCs induce angiogenesis by the development of the capillaries. Ischemia reduced the number of capillaries and large blood vessels but increased the number of small vessels and these changes were associated with reduced capillary to gastrocnemius muscle fiber ratio. Ischaemia-reduced capillary density and number of blood vessels in the region of the femoral artery transection were reversed in animals receiving MSCs and MCs. Furthermore, in the gastrocnemius muscle, immunohistochemical and histomorphometric observation showed a great ratio of capillaries to muscle fibers in all the cell-receiving groups. In contrast, there was little or no effect of the hydrogel scaffold alone on capillary density and number of vessels. This data supports the concept that tissue engineering with a hydrogel scaffold overcomes issues of cell engraftment seen previously^28,35^. Overall, the induction of angiogenesis by cell-based therapy in combination with the hydrogel scaffold was associated with increasing of distribution of collagen fibers and reduction of the capillary: gastrocnemius muscle fiber ratio.

Morphometric analysis of capillary angiogenesis showed the development of blood vessels in both MC- and MSC-treated groups. Interestingly, the enhancement of capillary density was seen mainly in the MC-treated group. In addition, the combination of a hydrogel scaffold with MCs showed significantly increases the muscle fiber diameter with an concomitant reduction in capillary-to gastrocnemius muscle fiber ratio. Angiogensis following any insult is regulated by the release of factors from endothelial cells^42,43^. One important stimuli is chronic hypoxia which leads to the secretion of growth factors from stromal or parenchymal cells including vascular cells. However, these factors alone were not sufficient to obtain complete repair of the local damage^43–45^.

Cellular therapies play an important role in angiogenesis^8,9^ and MSCs in particular can promote angiogenesis and arteriogenesis through stromal and paracrine actions^10,11^ especially during the treatment of injured tissues^14,46,47^. MSCs act, not only through the paracrine secretion of growth factors, but also induce the differentiation of endothelial cells, pericytes and myofibroblasts^45,48–50^.

MCs are activated in early phase after the induction of ischemia and mast cell degranulation occurs in the ischemic environment of the hind limb in the ICL model used here. However, the precise mechanism driving degranulation is largely unknown^51^. MCs do not differentiate into other cells, but secrete cytokines and other factors that enable the migration and differentiation of the other cells within the ischemic area^18^.

Emerging evidence indicates the relationship between angiogenesis, MSCs and MCs^17,18^. For example, MCs^17,18,51^ and MSCs^14,19^ are reported in or near the site of capillary germination which suggests a role of these cells in angiogenesis. MSCs have inhibitory effects on MCs that prevents their migration, degranulation and discharge^48,52^. Thus, it seems that the cross-talk between MSCs and MCs in the development of angiogenesis is very critical.The present study shows that the mean number of capillaries in the MC-treated group was similar to that in the MC + MSC-treated group but less than that in the MSC-treated group alone. However, the interaction was complex and varied according to the diameter of the vessel with small and large vessels showing some additional impact of both cells together whilst the effect of the combined cell treatment was inhibitory at intermediate sized vessels.

Despite these strengths of the study there were some limitations. We did not confirm the identity of the isolated mesenchymal cells following FACs analysis although this has been described by others^53–57^. In addition, it may have been useful to analyse tissue further from the implanted meshes to further understand the pathobiology. In addition, there are more modern acellular composite matrices that may represent better opportunities to improve cell therapy strategies for CLI particularly if they were initially assessed in large animal models compared to the smaller rat model used here.

## Conclusions

The results of current study indicated that bioengineered tissue incorporating MCs and MSCs within a hydrogel scaffold stimulates angiogenesis under ischemic conditions and lead to anastomosis in vessels. Both MCs and MSCs may prove important therapeutically in driving angiogenesis under conditions where critical limb ischemia has occured. However, further research in this area is required to determine the optimal combination of scaffold and cells that induce angiogenesis.
